# Human Leptospirosis Trends: Northeast Thailand, 2001–2012

**DOI:** 10.3390/ijerph110808542

**Published:** 2014-08-20

**Authors:** Wilawan Thipmontree, Yupin Suputtamongkol, Wiwit Tantibhedhyangkul, Chuanpit Suttinont, Ekkarat Wongswat, Saowaluk Silpasakorn

**Affiliations:** 1 Maharat Nakhon Ratchasima Hospital, Nakhon Ratchasima Province 30000, Thailand; E-Mails: noonmed@yahoo.com (W.T.); Chuanpit_s@hotmail.com (C.S.); 2 Department of Medicine, Faculty of Medicine Siriraj Hospital, Mahidol University, Bangkok 10700, Thailand; E-Mails: wiwit167@yahoo.co.th (W.T.); Dave_bio4@hotmail.com (E.W.); saowaluk_8@yahoo.com (S.S.)

**Keywords:** acute undifferentiated fever, leptospirosis, lung hemorrhage, Thailand

## Abstract

The objective of this study was to determine the changing trend of leptospirosis over time in Thailand using two prospective hospital-based studies conducted amongst adult patients with acute undifferentiated fever (AUFI) admitted to Maharat Nakhon Ratchasima Hospital, Nakhon Ratchasima Province, Thailand between July 2001 to December 2002 and between July 2011 to December 2012. During the first period, leptospirosis (98 patients, 40%) and scrub typhus (59 patients, 24.1%) were the two major causes of AUFI. In the second period, scrub typhus (137 patients, 28.3%) was found to be more common than leptospirosis (61 patients, 12.7%). Amongst patients with leptospirosis, the proportion of male patients and the median age were similar. *Leptospira interrogans* serogroup Autumnalis was the major infecting serogroup in both study periods. The case fatality rate of leptospirosis was significantly higher in 2011–2012 as compared with the case fatality rate in 2001–2002 (19.7% *vs.* 6.3%, *p* < 0.001). In summary, we found that number of leptospirosis cases had decreased over time. This trend is similar to reportable data for leptospirosis complied from passive surveillance by the Ministry of Public Health, Thailand. However, the case fatality rate of severe leptospirosis has increased. Severe lung hemorrhage associated with leptospirosis remained the major cause of death.

## 1. Introduction

Leptospirosis, caused by pathogenic members of genus *Leptospira*, is the most widespread bacterial zoonosis and a global public health problem [1]. A variety of wild and domestic animals excrete the organism in their urine, and human infection occurs through direct contact with infected animals or through exposure to fresh water or soil contaminated by infected animal urine. In Thailand reporting of cases of leptospirosis is mandatory, and since 1996 there has been a marked increase in the number of reported leptospirosis cases and leptospirosis-associated deaths occurring annually in this country [2]. The peak incidence period was seen during 2000–2003 after which the annual number of reported case decreased over time.

Leptospirosis may result in a subclinical manifestation, or may present as a self-limited anicteric febrile illness with or without meningitis or as a severe and potentially fatal illness known as Weil’s syndrome [3]. Severe pulmonary involvement and multi-organ failure have emerged as the main cause of death associated with leptospirosis in many countries, including Thailand [4,5]. The reported mortality rate of severe pulmonary hemorrhage syndrome caused by diffuse alveolar hemorrhage varied from 40% to 75% [4,5,6,7,8]. To determine changing trends of this disease over time in Thailand, we analyzed data from two clinical studies conducted in a tertiary hospital in North Eastern Thailand between July 2001 to December 2002, and between July 2011 to December 2012.

## 2. Material and Methods

### 2.1. Patients and Study Site

Maharat Nakhon Ratchasima Hospital is a 1000-bed government hospital located in Nakhon Ratchasima, a province in the northeast of Thailand with a population of 2,600,000. Most of the population work in farming, cultivating crops such as rice, sugar cane, tapioca, sesame and fruits and the rainy season lasts from late May to mid-October. Two prospective hospital-based studies have been conducted amongst adult patients with acute undifferentiated fever (AUFI) admitted to Maharat Nakhon Ratchasima Hospital. The first study, conducted between July 2001 and December 2002 was part of a multicenter study of the causes of acute, undifferentiated, febrile illness in rural Thailand [9]. Included in this study were adult patients (≥18 years) who presented with acute fever (oral temperature, ≥38.0 °C for <15 days) in the absence of an obvious focus of infection. Patient with malaria, and those with clinically obvious dengue-virus infection (who met the World Health Organization’s criteria for diagnosing dengue infection [10] were excluded. A second study was conducted between July 2011 and December 2012 using similar inclusion and exclusion criteria. Both study protocols were approved by the Ethical Review Subcommittee of the Public Health Ministry of Thailand and the Ethical Review Subcommittee of the Faculty of Medicine Siriraj Hospital, Mahidol University. Written inform consent was obtained from all study participants.

Data obtained from both studies were similar and included a detailed history and the findings of physical examinations and laboratory investigations which were recorded on a standard form. Investigations performed as baseline included a full blood cell and platelet count, 2 blood cultures for the detection of common aerobic bacteria, a leptospiral culture of 5 mL of blood collected in a sterile heparinized bottle and placed in Ellinghausen-McCullough medium as modified by Johnson and Harrison (EMJH), determination of plasma glucose, electrolyte, serum urea, and creatinine levels; liver function tests; urinalysis; and chest radiography. At least two plasma samples, which were obtained at admission and at an outpatient visit occurring at 2–4 weeks after discharge, were stored at −20 °C until serologic testing was performed. All plasma samples underwent serologic tests for the diagnosis of leptospirosis, scrub typhus, murine typhus, and dengue infection, as described below.

### 2.2. Diagnostic Tests

Diagnostic tests for leptospirosis included culture in EMJH medium, a serologic test for leptospirosis by indirect immunofluorescent antibody assay (IFA) and real-time quantitative polymerase chain reaction (qPCR) targeting the lipL32 gene [11]. PCR was performed on all plasma samples obtained during the second study and on plasma sample of patients with an unknown diagnosis during the first study. A definite diagnosis of leptospirosis was indicated by: (1) the isolation of leptospires or detection of leptospires DNA from blood; or (2) a 4-fold or greater increase in the specific IgG and IgM antibody titers (to ≥1:200; as determined by IFA); or (3) a single titer or stable antibody titer of ≥1:400 [12]. All leptospires isolated from both studies were confirmed and serotyped by the World Health Organization/Food and Agricultural Organization of the United Nations/Office International des Epizooties Collaborating Centre for Reference and Research on Leptospirosis (Brisbane, Australia). The criteria for the diagnosis of rickettsial infection (e.g., scrub typhus, murine typhus, and spotted fever group rickettsiosis) were: (1) at least a 4-fold increase in the specific anti-rickettsial IgG or IgM titer (to ≥1:400; as determined by IFA) between paired serum samples; or (2) a single titer or stable IFA titer of ≥1:400 [13].

Complications in patients with confirmed leptospirosis were prospectively classified according to organ-system involvement manifested by hypotension (*i.e.*, a systolic blood pressure of <90 mm Hg or a sustained decrease in systolic blood pressure of ≥40 mm Hg), jaundice (an increase in the total bilirubin level to ≥3 mg/dL), acute kidney injury or renal dysfunction (either oliguria (*i.e.*, a urine output of <0.5 mL/kg/h for at least 1 h) or azotemia (a serum creatinine level of ≥2.5 mg/dL), pulmonary involvement (abnormal findings on a chest radiograph or the need for mechanical ventilation at admission to the hospital), a decreased level of consciousness, or hemorrhagic complications (e.g., hemoptysis or gastrointestinal bleeding) or thrombocytopenia, defined as a platelet count of less than 100 × 10^6^ /mm^3^.

### 2.3. Statistical Analysis

Descriptive statistics were used to summarize the demographic data and baseline clinical characteristics. Differences between groups were analyzed using the χ^2^ test for categorized variables, and the *t*-test, for continuous variables. Patients with suspected co-infection of leptospirosis and another disease were excluded from the analysis.

## 3. Results

### 3.1. Period 1: July 2001–December 2002

A total of 245 patients with AUFI were enrolled in this study period and leptospirosis was diagnosed in 98 (40%) of them. The diagnosis of leptospirosis was made by isolation of leptospires from blood samples for five patients (one patient had both leptospires and *Burkholderia pseudomallei* detected in the blood sample and leptospirosis co-infection with scrub typhus was suspected in another patient), by positive PCR for eight patients, by a four-fold rise of antibody against leptospires by IFA for 51 patients and by a single titer or stable antibody titer of ≥1:400 for 36 patients. Overall 18 patients had suspected co-infection of leptospirosis with another infection (including two patients with blood culture positive for leptospires). Among patients with leptospirosis, 89.5% were male. The median age was 41 (range 18 to 75 years) and six (6.3%) patients died.

### 3.2. Period 2: July 2011–December 2012

**Figure 1:**
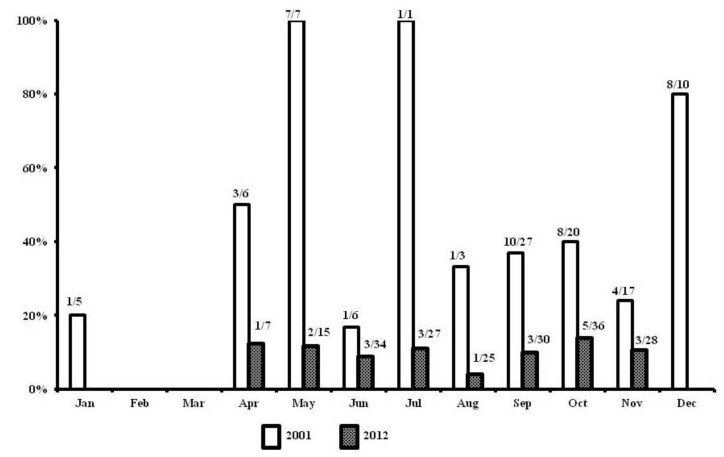
The proportion of leptospirosis amongst patients with acute undifferentiated fever in 2002 and 2012. (The figure above each bar represents the number of leptospirosis cases diagnosed among total number of AUFI).

A total of 481 patients were enrolled in this study period. Using similar inclusion and exclusion criteria, we found that proportion of male patients was significantly less than the first period. Scrub typhus (137 patients, 28.3%) was found to be the most common cause of AUFI in this study period. Demographic data and all causes of AUFI, compared between the two study periods, are shown in Table 1. Leptospirosis was diagnosed in 61 patients (12.7%). Diagnosis of leptospirosis was made by isolation of leptospires from blood for four patients, by DNA detection for 48 patients (including four patients with culture positive), by a four-fold rise of antibody for eight patients and by a single titer or stable antibody titer of ≥1:400 for five patients. *Leptospira interrogans* serogroup Autumnalis was identified as the major infecting serogroup in both study groups. However, leptospires isolated from a fatal case during the second study period was identified as a new serogroup worldwide. The proportion of patients with leptospirosis, diagnosed amongst patients with acute undifferentiated fever by month comparing between the years 2002 and 2012 is shown in Figure 1.

**Table 1:** Characteristics of AUFI patients in the two study periods.

Characters	2001–2002 (*n* = 245)	2011–2012 (*n* = 481)	*p*-value
Male, *n* (%)	199 (81.2)	319 (66.3)	<0.001
Mean (SD) age, years.	45 (16)	47 (18)	NS
Mean (SD) duration of fever, day	5.6 (3)	5.3 (2.9)	NS
Final diagnosis, *n* (%)			
Leptospirosis	98 (40)	61 (12.7)	
Scrub typhus	59 (24.1)	137 (28.5)	
Dengue infection	11 (4.5)	27 (5.6)	
Other diagnosis	8 (3.3)	34 (6.9)	
Co-infection *	18 (7.3)	9 (1.9)	
Unknown	51 (20.8)	213 (44.3)	
Overall mortality	22 (9)	50 (10.4)	NS

Note: * suspected co-infection of leptospirosis with another infection such as scrub typhus.

During the two study periods, the distribution of age and sex of patients with leptospirosis was similar (Table 2) although the clinical characteristics of leptospirosis have changed over the last ten years. A history of headache, cough, myalgia, calf pain and/or calf tenderness on admission showed a statistically significant difference between the two study periods in a bivariate analysis. Severe complications such as hypotension, pulmonary dysfunction, including pulmonary hemorrhage and multi-organ dysfunction were significantly more frequent in the study period 2011–2012 than in the study period 2001–2002, as shown in Table 2. As a result, significantly more patients required mechanical ventilator support during the second study period than in the first study period. Overall 12 patients died (10 males and two females) in the second period, and the mortality of leptospirosis was significantly higher in this period than in the period 2001–2002 (19.7% *vs.* 6.3%, *p* = 0.01).

**Table 2:** Characteristics of patients with leptospirosis.

Characters	2001–2002 (*n* = 98)	2011–2012 (*n* = 61)	*p*-value
Male:Female	87:11	54:7	NS
Mean (SD) age, years	42 (14)	46 (14)	0.037
Mean (SD) duration of fever, day	5 (2.4)	4 (1.4)	0.013
Headache	87 (88.8)	33 (54.1)	<0.001
Cough	73 (74.7)	21 (34.4)	<0.001
Myalgia	93 (94.9)	46 (75.4)	<0.001
Calf pain/tenderness	89 (90.8)	27 (44.3)	<0.001
Abnormal chest X-ray	34/88 (38.6)	42/61 (68.8)	0.001
Hypotension	14/98 (14.4)	37/61 (60.7%)	<0.001
Acute kidney injury	71 (72.4)	38 (62.3)	NS
Hepatic dysfunction			NS
None	24 (25.5)	21 (34.4)	
Hyperbilirubinemia	30 (31.9)	22 (36.1)	
Mixed type	35 (37.2)	15 (24.6)	
Pulmonary hemorrhage	9 (9.5)	20 (32.8)	0.001
Multi-organ dysfunction	27 (27.6)	31 (50.8)	0.004
Thrombocytopenia	78 (79.6)	50 (82)	NS
On mechanical ventilator	21 (21.4)	31/61 (51.8)	<0.001

### 3.3. Fatal Cases

All the cases of fatal leptospirosis had organ dysfunction on admission and 14 patients (77.8%) had multi-organ dysfunction on admission. Pulmonary hemorrhage occurred in eight patients (six of them associated with multi-organ dysfunction). The median duration of fever was 3 days (range 3–10) and the median duration of admission was 4 days (range 1–16). While the PCR test was positive in samples from all fatal patients, only five patients had detectable antibodies by IFA. The clinical presentation and the laboratory results comparing between fatal cases and patients who survived are shown in Table 3.

**Table 3:** Clinical characteristics and laboratory results compared between patients who survived and those who died.

Characters	Surviving (*n* = 141)	Deceased (*n* = 18)	*p*-value
Male, *n* (%)	125 (88.7)	16 (88.9)	NS
Duration of fever, day	4 (1–13)	3 (3–10)	NS
Hypotension/CVS involvement, *n* (%)	39 (27.9)	12 (66.7)	0.002
Abnormal LFT, *n* (%)	95 (69.3)	15 (83.3)	NS
Acute kidney injury, *n* (%)	95 (67.4)	14 (77.8)	NS
Pulmonary involvement, *n* (%)	36 (27.3)	12 (66.7)	0.002
Multi-organ dysfunction, *n* (%)	44 (31.2)	14 (77.8)	<0.001
Thrombocytopenia, *n* (%)	110 (78)	18 (100)	0.027
Laboratory results			
CBC, mean (SD)			
Hct, %	34.2 (5.2)	29.9 (4.8)	0.001
Total WBC, ×10^3^/cu.mm	11.63 (5.4)	13.25 (5.2)	NS
Blood chemistries, mean (SD)			
Total bilirubin, mg/dL	6.7 (7.3)	10.2 (7.9)	NS
AST, IU	272 (379)	106 (145)	<0.001
ALT, IU	78 (188)	96 (93)	NS
Alkaline phosphatase, IU	141 (102)	123 (65)	NS
Blood urea, mg/dL	53 (35.5)	59.5 (28.9)	NS
Serum creatinine, mg/dL	4.3 (2.8)	4.7 (2.1)	NS

## 4. Discussion and Conclusions

Although the same inclusion and exclusion criteria were used in both studies, the total number of AUFI patients in the second study period was almost double that of the first study period. The proportion of females was significantly increased in the second study period as compared to the first period. There is no clear explanation for this finding. In addition, the number of patients who remained with an unknown cause of AUFI was also very high in the second study period as compared to the first period. This finding indicated that AUFI was an important problem encounter in rural Thailand. As similar laboratory investigations were undertaken in both periods in order to obtain a diagnosis, the reason for the larger proportion of patients remaining with an unknown cause of AUFI in the second study period remains unknown.

Leptospirosis was found to be the most common cause of AUFI during the period 2001–2002, as shown in Figure 1, although the number of leptospirosis cases has decreased over time. This trend is similar to reportable data for leptospirosis complied from passive surveillance by the Ministry of Public Health, Thailand. There has been no obvious environmental change and no implementation of human or animal vaccine for the prevention of leptospirosis Thailand. However after the initial outbreak of leptospirosis, a mass education campaign has been implemented in the country to raise the awareness of the population about leptospirosis. There has been also an increased awareness among physicians in Thailand that AUFI is a common presentation of leptospirosis and scrub typhus. After the outbreak of leptospirosis in Thailand, doxycycline or oral azithromycin has been widely used as an empirical antimicrobial therapy for mild AUFI in adult and non-pregnant woman. Ceftriaxone has been increasing used as an initial empirical antimicrobial treatment for patients with suspected severe bacterial infection or community-acquired sepsis in Thailand too.

The reported incidence of acute kidney injury or renal dysfunction, pulmonary involvement, hypotension or cardiovascular manifestation associated with severe leptospirosis varies widely in the literature, according to the definition of organ dysfunction and inclusion criteria used [14,15]. Maharat Nakhon Ratchasima Hospital is a tertiary hospital and therefore nearly all of the patients with laboratory confirmed leptospirosis in this study had severe manifestations. However, the results of this study showed that the clinical characteristics of severe leptospirosis may be changing over time, since the presence of headache, myalgia, calf pain and/or tenderness were less frequent, but organ dysfunction especially cardiac dysfunction, pulmonary involvement and multi-organ dysfunction were more frequent in the more recent period (2011–2012) when compared with the initial outbreak period (2001–2002). Despite prompt antimicrobial treatment with ceftriaxone, the case fatality rate of leptospirosis was found to be significantly higher in the period 2011–2012 as compared to the period 2001–2002, and severe lung hemorrhage associated with multi-organ failure was the major cause of death. We applied the same definition of severe complications of leptospirosis in these two studies. The mortalities among patients with other causes of AUFI, such as severe scrub typhus, or sepsis from other bacteria, compared between the second and the first study period, were similar or slightly decreased (but not statistically significant different, data not shown here). In addition the mean duration of fever at admission amongst patients with AUFI in the two studies was similar, but amongst patient with leptospirosis, it was significantly shorter in the second period than in the initial period. Although patients with leptospirosis from 2011–2012 were significantly older than patients of the 2001–2002, their mean ages were within the same decade, and the mean age of patients who survived and who died were similar (data not shown here). Therefore higher mortality in the second period was not explained by the older age and delay of medical treatment. The higher proportion of severe manifestations or severe complications found during the second study period might be one explanation for the higher mortality of leptospirosis in this period. Although *L. interrogans* serogroup Autumnalis remains the major type of leptospires that we were able to isolate from patients with leptospirosis, only four patients with positive blood culture were found in the second period. A change in the infective serotype of leptospires in this area is one possible explanation for the changing clinical manifestations. This is supported by the isolation of one new serotype amongst the four isolates, and the new serotype was isolated from a fatal case. More effort in the cultivation of leptospires or in the application of advance molecular techniques to identify genotype and serotype of infective leptospires causing human leptospirosis in this area is needed.

Because of its protean clinical manifestations, leptospirosis mimics many other infectious diseases. However, another important finding from this study is that most of the fatal cases of leptospirosis were diagnosed by PCR while specific antibodies were detectable in only 5 out of 18 cases. This suggests that the case fatality rate of leptospirosis would be very much underestimated in the absence of PCR analysis.

In conclusion the number of cases of leptospirosis has decreased over time, however the clinical characteristics have changed and the case fatality rate of severe leptospirosis has increased. Severe lung hemorrhage associated with leptospirosis remains the major cause of death.
